# Studies directed toward the exploitation of vicinal diols in the synthesis of (+)-nebivolol intermediates

**DOI:** 10.3762/bjoc.13.56

**Published:** 2017-03-21

**Authors:** Runjun Devi, Sajal Kumar Das

**Affiliations:** 1 Department of Chemical Sciences, Tezpur University, Napaam, Tezpur, Assam-784028, India

**Keywords:** dihydroxylation, epoxide-ring opening, heterocycles, nebivolol, *syn*-epoxide

## Abstract

While the exploitation of the Sharpless asymmetric dihydroxylation as the source of chirality in the synthesis of acyclic molecules and saturated heterocycles has been tremendous, its synthetic utility toward chiral benzo-annulated heterocycles is relatively limited. Thus, in the search for wider applications of Sharpless asymmetric dihydroxylation-derived diols for the synthesis of benzo-annulated heterocycles, we report herein our studies in the asymmetric synthesis of (*R*)-1-((*R*)-6-fluorochroman-2-yl)ethane-1,2-diol, (*R*)-1-((*S*)-6-fluorochroman-2-yl)ethane-1,2-diol and (*S*)-6-fluoro-2-((R)-oxiran-2-yl)chroman, which have been used as late-stage intermediates for the asymmetric synthesis of the antihypertensive drug (*S*,*R*,*R*,*R*)-nebivolol. Noteworthy is that a large number of racemic and asymmetric syntheses of nebivolol and their intermediates have been described in the literature, however, the Sharpless asymmetric dihydroxylation has never been employed as the sole source of chirality for this purpose.

## Findings

**Figure 1:**
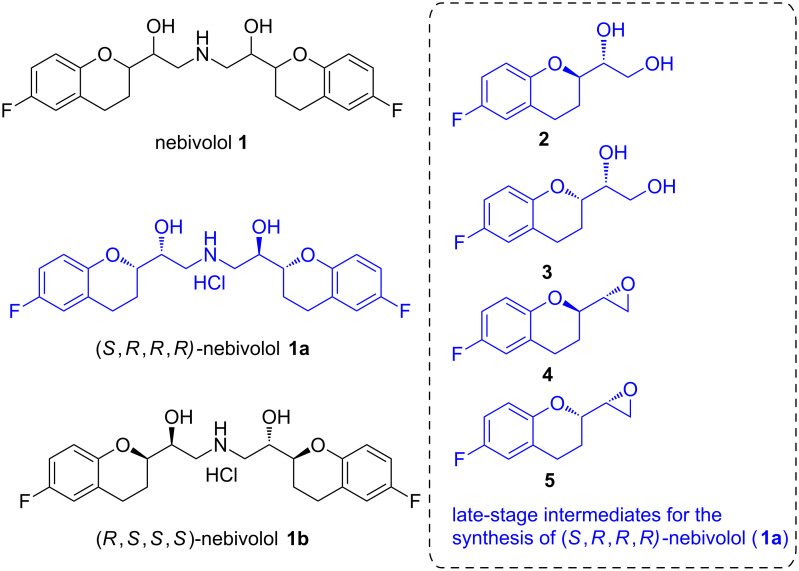
The chroman-based antihypertensive drug nebivolol, its biologically active stereoisomers and late-stage intermediates for its synthesis.

Chiral chromans are prevalent components of a large number of natural products, natural product-like molecules and pharmaceutical drugs, possessing diverse biological activities [1]. In view of their wide spectrum of biological profiles, chiral chromans have become attractive synthetic targets in academia and pharmaceutical industry [1]. Nebivolol (**1**, Figure 1) is a chroman-based antihypertensive drug that was first reported in the racemic form [2–3]. Chiral HPLC was subsequently employed to access various stereoisomers of **1** in enantiomerically pure form [4–5]. Out of the ten possible stereoisomers of **1**, (*S*,*R*,*R*,*R*)-nebivolol or (+)-nebivolol (**1a**, Figure 1) was found to be a potent β_1_-adrenergic receptor blocker [2–3]. On the other hand, the corresponding enantiomeric form, (*R*,*S*,*S*,*S*)-nebivolol or (−)-nebivolol (**1b**, Figure 1) was found to be devoid of β_1_-antagonist activity, but it showed a significant synergistic effect on the antihypertensive proficiency of the (*S*,*R*,*R*,*R*) isomer [6–9].

**Scheme 1:**
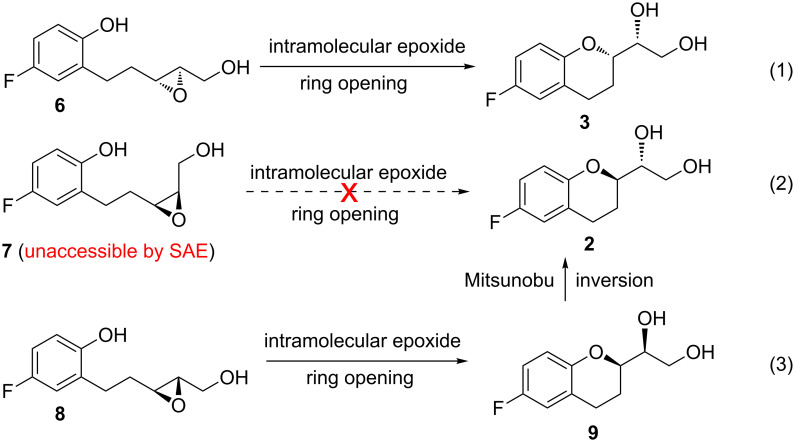
Synthetic strategies toward late-stage intermediates of **1a**.

A number of syntheses of nebivolol and its intermediates have been described in the literature [10–19]. Most of these have demonstrated that the synthesis of **1a** could be achieved using the 2-substituted chroman derivatives (*R*)-1-((*R*)-6-fluorochroman-2-yl)ethane-1,2-diol (**2**) and (*R*)-1-((*S*)-6-fluorochroman-2-yl)ethane-1,2-diol (**3**) or the corresponding chroman epoxides **4** and **5** as late-stage intermediates. Although the consensus synthetic strategy for **1a** involves the convergent assembly of chroman-based key subunits, the question of how best to access them remains open. The intramolecular ring-opening of enantiomerically pure epoxides by the phenolic hydroxy group is one of the most popular methods to construct **3** (Scheme 1, method 1). For this purpose, the necessary epoxide **6** could be obtained from the parent *E*-allylic alcohol through Sharpless asymmetric epoxidation (SAE) [11]. However, the corresponding parent *Z*-allylic alcohol appears to be not suitable to provide **7** under SAE conditions [20]. This has eliminated the possibility of obtaining **2** via intramolecular epoxide ring-opening of **7** (Scheme 1, method 2). Consequently, an alternative pathway involving the Mitsunobu inversion of **9** (obtained by intramolecular epoxide ring-opening of **8** which is the enantiomer of **6**) has been followed to obtain **2** (Scheme 1, method 3) whereupon the overall yield of the reaction sequence diminished [11].

On the other hand, the Sharpless asymmetric dihydroxylation (SAD) has been a workhorse as a synthetic tool for accessing enantiopure vicinal diols [21]. The extensive work in this field has resulted in the discovery of a number of cinchona alkaloid-derived ligands which allow the dihydroxylation of alkenes of almost all substitution patterns with high enantioselectivity. Noteworthy is that the SAD is not limited to only *E*-allylic alcohols in its choice of substrates as is the SAE process. Moreover, the SAD is much more superior in terms of operational simplicity as SAE, it can be run at 0 °C in water as a co-solvent and under an atmosphere open to air.

The application of SAD-derived vicinal diols in the synthesis of acyclic molecules and saturated heterocycles has been astonishing. However, their utilities in the synthesis of chiral benzo-annulated heterocycles are relatively limited [22–26]. In this paper, we describe our efforts toward the synthesis of **2**, **3** and **5** using different cyclization strategies. To the best of our knowledge, nebivolol or its intermediates have never been synthesized using Sharpless asymmetric dihydroxylation as the sole source of chirality [27].

**Scheme 2:**
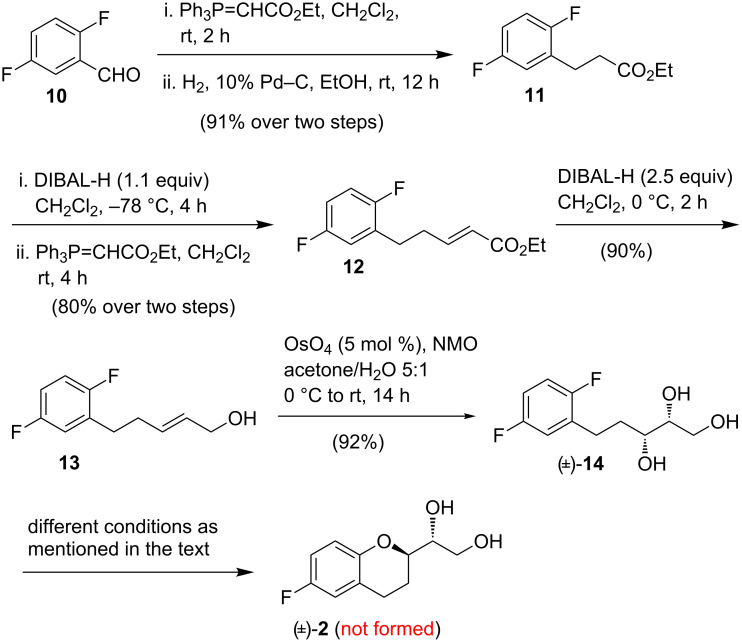
Attempted synthesis of (±)-**2** via intramolecular S_N_Ar reaction.

For the synthesis of chroman derivative **2**, first a base-mediated intramolecular S_N_Ar reaction was envisioned for the aryl C–O bond formation under transition-metal-free conditions [28–30]. The additional benefit of this strategy would be the non-requirement of any protecting group to construct the chroman ring. To test this seemingly straightforward approach, we initially attempted to synthesize (±)-**2** utilizing the cyclization of (±)-triol **14** (Scheme 2) as the key step.

Thus, commercially available 2,5-difluorobenzaldehyde (**10**) was treated with the Wittig reagent Ph_3_P=CHCO_2_Et and the resulting unsaturated ester was then hydrogenated with Pd–C and H_2_ at room temperature to obtain compound **11** in 91% yield over two steps (Scheme 2). DIBAL-H (1.1 equiv, –78 °C) reduction of the ester group of **11** followed by Wittig olefination of the resulting crude aldehyde with Ph_3_P=CHCO_2_Et provided (*E*)-α,β-unsaturated ester **12** (80% over two steps). A further DIBAL-H (2.5 equiv, 0 °C) reduction of **12** delivered (*E*)-allylic alcohol **13** (90%) which was then dihydroxylated under Upjohn conditions to obtain triol (±)-**14** (92%). With access to (±)-**14** we were in a position to investigate the key cyclization involving an intramolecular S_N_Ar to deliver **2**. Unfortunately, all attempts of cyclizing (±)-**14** to obtain chroman derivative (±)-**2** under various S_N_Ar reaction conditions were not successful. Treatment of (±)-**14** with KO*t-*Bu/THF (65 °C), NaH/DMF (80 °C), NaH/DMSO (100 °C) and KO*t-*Bu/toluene (110 °C) did not lead to any conversion. However, more forcing conditions such as NaH/NMP (130 °C) resulted in a to partial decomposition of the starting material. These results indicated that an intramolecular S_N_Ar reaction of triol (±)-**14** to form (±)-**2** is not feasible. Thus, the presence of an activating substituent (e.g., a nitro group) at the C-5 position of the benzene ring might be helpful in synthesizing molecules similar to **2** [31–32].

**Scheme 3:**
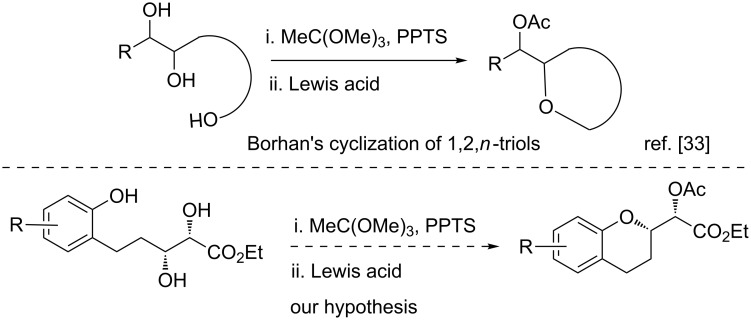
Speculation on the synthesis of a 2-substituted chroman derivative based on Borhan’s approach.

The failure to achieve an intramolecular cyclization of the diol via an S_N_Ar reaction caused us to investigate other cyclization approaches towards these chroman derivatives. In 2005, Borhan and co-workers described the construction of tetrahydrofuran and tetrahydropyran structures from 1,2,*n*-triols via an elegant cyclization involving Lewis acid-mediated cyclization of in situ generated cyclic orthoesters [33]. We speculated that a similar reaction on an appropriately positioned diol with a tethered *o*-hydroxyphenyl group might produce 2-substituted chroman derivatives via a 6-*exo*-*tet* cyclization (Scheme 3).

**Scheme 4:**
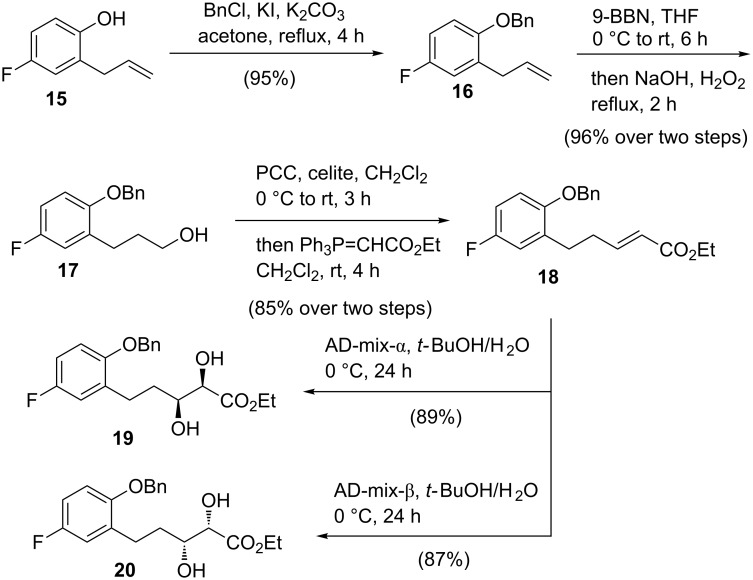
Synthesis of *syn*-2,3-dihydroxy esters **19** and **20**.

To substantiate this hitherto unexplored approach in the context of synthesizing (+)-nebivolol intermediates, we first needed to synthesize the *syn*-dihydroxy esters **19** and **20** (Scheme 4). Toward that objective, 2-allyl-4-fluorophenol (**15**) was benzylated with BnCl and anhydrous K_2_CO_3_ in the presence of KI in acetone under reflux conditions to obtain benzyl ether **16** (Scheme 4). The subsequent hydroboration of the allyl group in **16** with 9-BBN and the oxidation of the resulting organoborane with NaOH and H_2_O_2_ furnished alcohol **17** in 96% yield. A one-pot PCC oxidation–Wittig olefination (with Ph_3_P=CHCO_2_Et) of **17** provided (*E*)-α,β-unsaturated ester **18** in 85% yield over 2 steps. Compound **18** was then subjected to a Sharpless asymmetric dihydroxylation with AD-mix-α in *t*-BuOH/H_2_O (1:1) at 0 °C for 24 h furnishing *syn*-2,3-dihydroxy ester **19** in a high yield of 92%. For the synthesis of *syn*-2,3-dihydroxy ester **20**, AD-mix-β was employed.

**Scheme 5:**
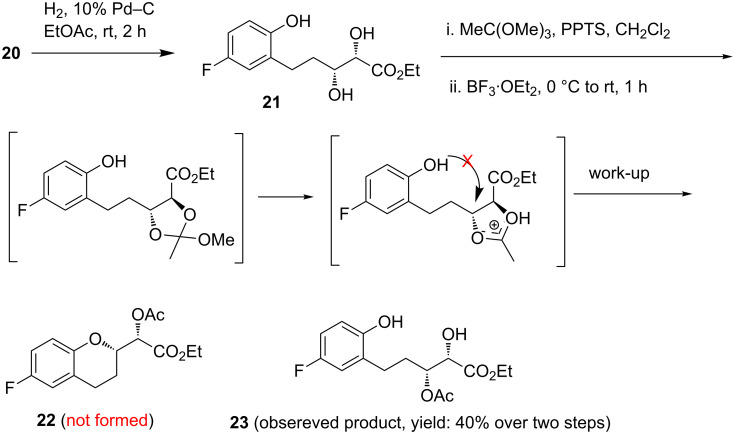
Attempted cyclization according to Borhan’s method.

Debenzylation of diol **20** with Pd–C and H_2_ at room temperature produced compound **21** having a tethered *o*-hydroxyphenyl group (Scheme 5). Next, compound **21** was exposed to Borhan’s reaction conditions with the hope to obtain **22**. To our dismay, however, the reaction furnished the hydrolyzed product **23** instead of cyclized product **22**. It is important to mention that strict anhydrous conditions were maintained for this transformation in order to prevent the nucleophilic attack of H_2_O leading to the formation of **23**. However, the formation of **23** instead of **22** clearly indicates that the in situ generated cyclic orthoester, after getting activated by Lewis acid, did not experience a nucleophilic attack of the phenolic hydroxy group; instead it reacted with water during the work-up process. This different outcome of this reaction compared to Borhan’s results might be attributed to the lower nucleophilicity of phenols compared to alcohols.

**Scheme 6:**
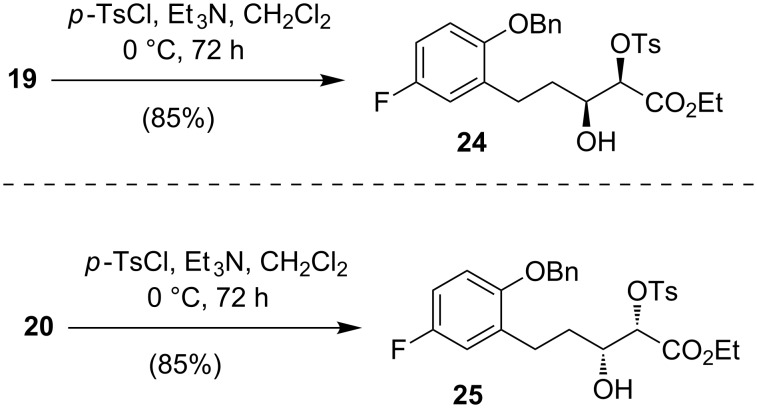
Synthesis of β-hydroxy-α-tosyloxy esters **24** and **25**.

Previously, Panda and co-worker converted succesfully a *syn*-2,3-dihydroxy ester into a 2-substituted chroman derivative [23]. The above-described unfortunate failures eventually forced us to turn our attention to utilize this methodology for the synthesis of 2-substituted chroman derivatives. Thus, diols **19** and **20** were subjected to a monotosylation reaction [34] to obtain the β-hydroxy-α-tosyloxy esters **24** and **25**, respectively (Scheme 6).

**Scheme 7:**
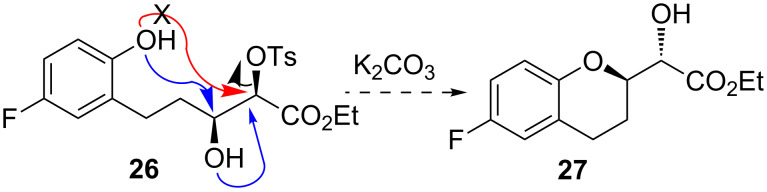
Speculation of simultaneous epoxidation/epoxide-ring opening.

Panda and co-worker applied a three-step reaction sequence involving epoxidation/debenzylation/epoxide ring-opening to convert the β-hydroxy-α-tosyloxy ester into the corresponding 2-substituted chroman derivative. However, it has been reviewed that not only benzylic epoxides but also non-benzylic epoxides are sensitive to the standard hydrogenation/debenzylation conditions [35]. Whereas benzylic epoxides are highly sensitive to hydrogenation conditions, non-benzylic epoxides, depending on the reaction conditions, may produce traces to significant amounts of side-products via hydrogenolysis. Thus, we decided to modify Panda’s synthetic route to significantly increase the overall yield. We hypothesized that this problem might be circumvented by performing the debenzylation reaction prior to the epoxide-ring formation. Further we speculated that compound **26**, in the presence of a base, might undergo a simultaneous epoxidation–intramolecular epoxide-ring opening to produce **27** (Scheme 7) as the corresponding benzoxepin ring formation via intramolecular displacement of –OTs group by ArO^−^ is unresponsive [23].

**Scheme 8:**
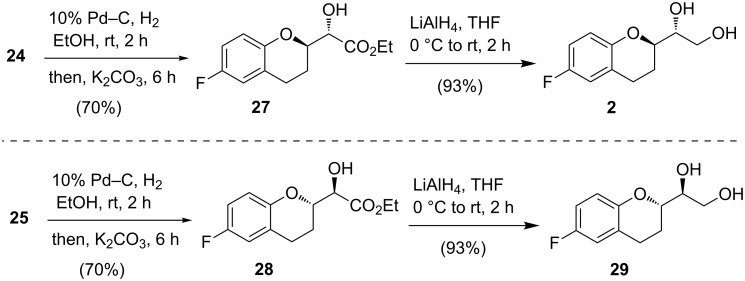
Synthesis of chroman diols **2** and **29**, respectively.

**Scheme 9:**
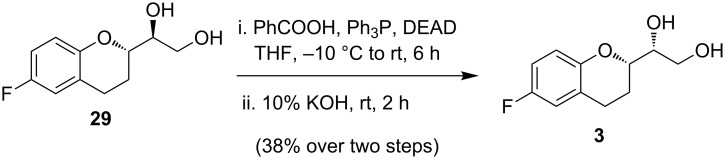
Conversion of **32** into **3** via Mitsunobu inversion.

To test this hypothesis, first compound **24** was subjected to the debenzylation reaction with 10% Pd–C in EtOH under an H_2_ atmosphere (Scheme 8) at room temperature. After completion of the debenzylation process, K_2_CO_3_ was added to the reaction mixture in the same reaction vessel. The reaction mixture, after being run for additional 6 h, provided compound **27** in 70% yield which is significantly higher than the literature yield (53%) for the similar transformation [23]. The similar strategy was applied in converting **25** into chroman derivative **28**. Next, LiAlH_4_ reduction of **27** and **28** provided **2** and **29**, respectively, in 93% yield. It is to be mention that the NMR spectra and specific rotations of **2** and **29** matched with those reported in the literature [11,14–15]. For the synthesis of compound **3**, stereoisomer **29** was subjected to classical two-step Mitsunobu inversion protocol which was successful but poor yielding (Scheme 9).

**Scheme 10:**
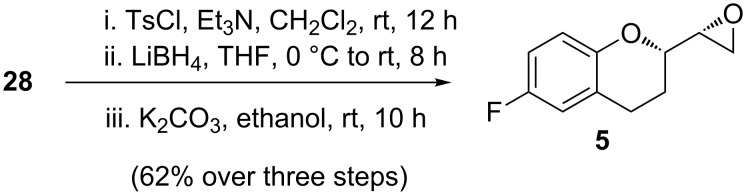
Synthesis of chroman epoxide **5**.

Not surprised by the poor yield of this transformation, we focused on the conversion of **28** into **5** which has also been used as a late-stage nebivolol intermediate (**5** is a more advanced intermediate compared to **3**). Thus, the tosylation of compound **28** followed by LiBH_4_ reduction of the resulting tosylate and subsequent epoxidation of the obtained tosyloxy alcohol with anhydrous K_2_CO_3_ in absolute ethanol (Scheme 10) provided compound **5**.

The NMR spectra and specific rotations of **5** also matched those reported in the literature [17–19,36].

## Conclusion

In summary, in the context of exploiting Sharpless asymmetric dihydroxylation-derived vicinal diols in the synthesis of (*R*)-1-((*R*)-6-fluorochroman-2-yl)ethane-1,2-diol, (*R*)-1-((*S*)-6-fluorochroman-2-yl)ethane-1,2-diol and (*S*)-6-fluoro-2-((*R*)-oxiran-2-yl)chroman, which have previously utilized as late-stage intermediates for the synthesis of (*S*,*R*,*R*,*R*)-nebivolol, we have extensively studied different cyclization strategies. The construction of 2-substituted chroman derivatives using phenolic hydroxy-mediated intramolecular ring opening of *syn*-2,3-diol ester-derived cyclic orthoester or intramolecular S_N_Ar reaction of a triol containing a tethered 2,5-difluorophenyl substituent were not successful. However, the exposure of β-hydroxy-α-tosyloxy esters to a one-pot, three-step process (debenzylation–epoxidation–intramolecular epoxide ring opening) enabled us to acheive the target molecules. To the best of our knowledge, this is the first use of the Sharpless asymmetric dihydroxylation as the sole source of chirality for the synthesis of nebivolol intermediates.

## Supporting Information

File 1 Experimental procedures, characterization data and copies of ^1^H and ^13^C NMR spectra for final compounds are available.
