# Supplementary material for: Studies directed toward the exploitation of vicinal diols in the synthesis of (+)-nebivolol intermediates
Source: Beilstein J Org Chem. 2017 Mar 21;13:571–8. doi: 10.3762/bjoc.13.56 (PMC5372751; doi:10.3762/bjoc.13.56)
Supplement: File 1 — Experimental procedures, characterization data and copies of 1H and 13C NMR spectra for final compounds are available. [file Beilstein_J_Org_Chem-13-571-s001.pdf]

Supporting Information

for

Studies directed toward the exploitation of vicinal diols in the synthesis of (+)-nebivolol intermediates

Runjun Devi and Sajal Kumar Das*

Address: Department of Chemical Sciences, Tezpur University, Napaam, Tezpur, Assam-784028, India

Email: Sajal Kumar Das* - sajalkdas@gmail.com

*Corresponding author

Experimental procedures, characterization data and copies of ^{1}H and ^{13}C NMR spectra for final compounds are available

Table of Contents

1. **General information**………………………………………………………………………………… **S2**
2. **Preparation of compounds**……………………………………………………………………………… **S2**
3. **References**………………………………………………………………………………………………….. **S10**
4. **Copies of ^{1}H and ^{13}C NMR spectra of compounds**……………………………………………….. **S11**

1. General information

All dry reactions were carried out under nitrogen in oven-dried glassware using standard gas-light syringes, cannulas, and septa. Commercial reagents were used without further purification unless otherwise stated. Progress of reactions was monitored by TLC on pre-coated Merck silica gel plates (60F-254). Visualization of reactants and products was accomplished with UV light. Column chromatography was performed over silica gel (60–120 mesh) procured from Merck using freshly distilled solvents. Melting points were determined with a Büchi-535 apparatus and are not corrected. Specific rotations were measured using a Rudolph Autopol-V polarimeter. A Perkin Elmer 20 analyzer was utilized for elemental analysis of all compounds. ^{1}H NMR and ^{13}C NMR spectra were run on a JEOL 400 MHz spectrometer in $CDCl_3$ as solvent. Tetramethylsilane (0.00 ppm) served as an internal standard in ^{1}H NMR and $CDCl_3$ (77.0 ppm) in ^{13}C NMR. All spectra were recorded at 25 °C. Coupling constants (*J* values) are given in hertz (Hz). Chemical shifts are expressed in parts per million (ppm).

2. Preparation of compounds

Ethyl 3-(2,5-difluorophenyl)propanoate (11) [1]

To a stirred solution of 2,5-difluorobenzaldehyde (**10**, 3.0 g, 21.11 mmol) in dichloromethane (100 mL) was added (carbethoxymethylene)triphenylphosphorane (9.2 g, 26.39 mmol) at room temperature. After stirring at room temperature for 2 h, the reaction mixture was concentrated in a rotatory evaporator. The resulting residue was suspended by adding 100 mL of 10% EtOAc in hexane and stirred vigorously. The mixture was then filtered and the filtrate was concentrated in a rotatory evaporator to get crude (*E*)-ethyl 3-(2,5-difluorophenyl)acrylate as light yellow oil which was used for the next step without further purification.

A suspension of 10% Pd–C (200 mg) and the above crude material in ethanol (50 mL) was stirred for 12 h at room temperature under pressure of a hydrogen balloon. The reaction mixture was filtered through a pad of Celite® and the filter pad was well-washed with EtOAc. The filtrates were combined and concentrated in a rotatory evaporator. The residue was purified by silica gel column chromatography (2% EtOAc in hexane) to afford **11** (4.12 g, 91% over two steps) as a colorless semi-solid. ^{1}H NMR (400 MHz, $CDCl_3$): δ 6.95-6.90 (m, 2H), 6.86-6.83 (m, 1H), 4.12 (q, *J* = 7.2 Hz, 2H), 2.93 (t, *J* = 7.6 Hz, 2H), 2.60 (t, *J* = 7.6 Hz, 2H), 1.23 (t, *J* = 7.2 Hz, 3H).

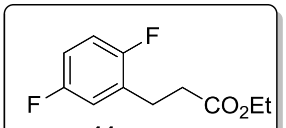

11

(*E*)-Ethyl 5-(2,5-difluorophenyl)pent-2-enoate (12)

DIBAL-H (20 mL, 20.0 mmol, 1 M in hexane) was added dropwise to a stirred solution of **11** (4.0 g, 18.67 mmol) in CH_2Cl_2 (50 mL) at −78 °C. The mixture was stirred for 4 h at −78 °C. The reaction was quenched at −78 °C by the slow addition of methanol (2 mL) and then warmed to rt. A saturated solution of Rochelle's salt (50 mL) was added and the resulting biphasic mixture was stirred vigorously for 1.5 h. After separating the organic layer, the aqueous phase was extracted with CH_2Cl_2 (2 × 50 mL) and the combined organic phases were dried over $MgSO_4$, filtered and concentrated in a rotary evaporator to get the crude aldehyde as a colorless gum which was used for the next step without further purification.

To a stirred solution of the above crude aldehyde in dichloromethane (200 mL) was added (carbethoxymethylene)triphenylphosphorane (6.97 g, 20.0 mmol) at rt. After stirring at room temperature for 4 h, the reaction mixture was concentrated in a rotatory evaporator. The resulting residue was suspended by adding 100 mL of 10% EtOAc in hexane and stirred vigorously. The mixture was then filtered and the filtrate was concentrated in a rotatory evaporator. Purification of the resulting residue by silica gel column chromatography (5% ethyl acetate in hexane) afforded compound **12** (3.59 g, 80% over two steps) as a light yellow oil. ^{1}H NMR (400 MHz, $CDCl_3$): δ 7.00-6.94 (m, 2H), 6.89-6.85 (m, 2H), 5.85 (dt, 1H, *J* = 15.8, 1.5 Hz), 4.19 (q, 2H, *J* = 7.2 Hz), 2.78 (t, 2H, *J* = 7.6 Hz), 2.53-2.48 (m, 2H), 1.28 (t, 3H, *J* = 7.1 Hz). ^{13}C NMR (100 MHz, $CDCl_3$): δ 166.4, 158.5 (d, J_{C-F} = 239.8), 156.9 (d, J_{C-F} = 237.9), 147.0, 129.2 (dd, J_{C-F} = 18.2 and 7.3 Hz), 122.2, 116.7 (dd, J_{C-F} = 23.6 and 3.6 Hz), 116.2 (dd, J_{C-F} = 25.4 and 7.3 Hz), 114.1 (dd, J_{C-F} = 23.6 and 9.1 Hz), 60.2, 32.1, 27.6, 14.2. Anal. Calcd. for $C_{13}H_{14}F_2O_2$: C, 64.99; H, 5.87. Found: C, 65.12; H, 5.93.

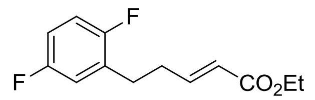

12

(*E*)-5-(2,5-Difluorophenyl)pent-2-en-1-ol (13)

DIBAL-H (15.0 mL, 15.0 mmol, 1 M in hexane) was added dropwise to a solution of **12** (1.5 g, 6.24 mmol) in CH_2Cl_2 (20 mL) at 0 °C. The mixture was stirred for 2 h at 0 °C. The reaction was quenched by adding a saturated solution of Rochelle's salt (30 mL) and the resulting biphasic mixture was stirred vigorously for 1 h. After separating the organic layer, the aqueous phase was extracted with CH_2Cl_2 (2 × 50 mL) and the combined organic phases were washed with brine, dried over $MgSO_4$, filtered and concentrated in a rotary evaporator. The residue was purified by silica gel column chromatography (20% ethyl acetate in hexane) to afford **13** (1.11 g, 90%) as a colorless gum. ^{1}H NMR (400 MHz, $CDCl_3$): δ 6.98-6.93 (m, 2H), 6.89-6.82 (m, 1H), 5.75-5.64 (m, 2H), 4.09 (t, 2 H, *J* = 5.5 Hz), 2.71 (t, 2 H, *J* = 7.6 Hz), 2.38-2.33 (m, 2 H), 1.38 (br s, 1 H). ^{13}C{^{1}H} NMR (100 MHz,$CDCl_3$): δ 158.5 (d, J_{C-F} = 243.4), 157.0 (d, J_{C-F} = 239.7), 131.2 (2C), 130.1 (dd, J_{C-F} = 19.9 and 7.2 Hz), 116.7 (dd, J_{C-F} =

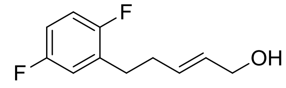

13

23.6 and 5.5 Hz), 116.0 (dd, J_{C-F} = 25.4 and 9.1 Hz), 113.7 (dd, J_{C-F} = 25.4 and 9.1 Hz), 63.5, 32.3, 28.6. Anal. Calcd. for $C_{11}H_{12}F_2O$: C, 66.66; H, 6.10. Found: C, 66.73; H, 6.24.

(±)-*threo*-5-(2,5-Difluorophenyl)pentane-1,2,3-triol (14)

To a solution of compound **13** (1.0 g, 5.04 mmol) and NMO (1.46 g, 12.5 mmol) in 60 mL acetone-H_2O (5:1) at 0 °C, was added OsO_4 (64 mg, 0.25 mmol) and the reaction mixture was stirred vigorously at the same temperature for 2 h and at rt for 12 h. The reaction was quenched with sodium bisulfite (1.0 g), diluted with water (100 mL) and extracted with ethyl acetate (3 × 50 mL). The combined organic layers were washed with brine, dried over $MgSO_4$, filtered and concentrated in a rotary evaporator. The residue was purified by silica gel column chromatography (60% ethyl acetate in hexane) to afford **14** (1.08 g, 92%) as a colorless solid. M.P.: 68-69 °C. ^{1}H NMR (400 MHz, $CDCl_3$): δ 6.94-6.89 (m, 2H), 6.84-6.80 (m, 1H), 3.89 (br s, 1H), 3.75-3.55 (m, 5H), 2.84-2.63 (m, 2H), 2.46 (br s, 1H), 1.83-1.72 (m, 2 H). ^{13}C{^{1}H} NMR (100 MHz,$CDCl_3$): δ 158.5 (d, J_{C-F} = 241.6), 157.0 (d, J_{C-F} = 239.8), 130.2 (dd, J_{C-F} = 18.1 and 7.3 Hz), 116.8 (dd, J_{C-F} = 23.6 and 5.5 Hz), 116.1 (dd, J_{C-F} = 25.4 and 7.3 Hz), 113.8 (dd, J_{C-F} = 23.6 and 9.1 Hz), 73.8, 71.6, 64.6, 33.5, 25.1. Anal. Calcd. for $C_{11}H_{14}F_2O_3$: C, 56.89; H, 6.08. Found: C, 56.96; H, 6.12.

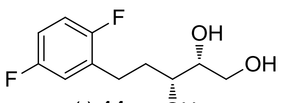

(±)-**14**

Attempted cyclization of (±)-*threo*-5-(2,5-Difluorophenyl)pentane-1,2,3-triol (14)

entry	reaction conditions	result
1	**14** (0.1 mmol), KO*t*Bu (4 equiv.), THF, 65 °C, 48 h	recovery of **14**
2	**14** (0.1 mmol), NaH (4 equiv.), DMF, 80 °C, 48 h	recovery of **14**
3	**14** (0.1 mmol), NaH (4 equiv.), DMSO, 100 °C, 48 h	recovery of **14**
4	**14** (0.1 mmol), KO*t*Bu (4 equiv.), toluene, 80 °C, 48 h	recovery of **14**
5	**14** (0.1 mmol), NaH (4 equiv.), NMP, 130 °C, 48 h	partial recovery of **14**

2-Allyl-1-(benzyloxy)-4-fluorobenzene (16) [2]

To a solution 2-allyl-4-fluorophenol (**15**, 10.0 g, 65.71 mmol) in dry acetone (150 mL) was added anhydrous K_2CO_3 (14.01 g, 101.59 mmol), benzyl chloride (7.58 mL, 65.71 mmol) and KI (500 mg). The reaction mixture was refluxed for 4 h. It was then concentrated in a rotary evaporator to remove acetone. The resulting residue was re-dissolved in ethyl acetate (200 mL) and water (200 mL). The organic layer was separated, washed with brine (100 mL), dried over anhydrous Na_2SO_4 and filtered. Solvent was removed in a rotary evaporator and the crude product was purified by silica gel column chromatography

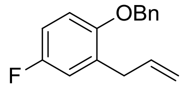

16

(2% ethyl acetate in hexane) to afford **16** (15.12 g, 95%) as a light yellow liquid. ^{1}H NMR (400 MHz, $CDCl_3$): δ 7.49-7.35 (m, 5H), 6.97-6.84 (m, 3H), 6.08-5.98 (m, 1H), 5.16-5.11 (m, 2H), 5.08 (s, 2H), 3.47 (d, 1H, J = 6.4 Hz). ^{13}C{^{1}H} NMR (100 MHz,$CDCl_3$): δ 157.1 (d, J_{C-F} = 238.6), 152.3, 137.0, 136.0,130.9 (d, J_{C-F} = 6.7), 128.5, 127.8, 127.1, 116.5 (d, J_{C-F} = 23.9), 116.2, 112.8 (d, J_{C-F} = 23.0), 112.6 (d, J_{C-F} = 7.6), 70.5 (t, J_{C-F} = 2.9), 34.2. Anal. Calcd. for $C_{16}H_{15}FO$: C, 79.32; H, 6.24. Found: C, 79.28; H, 6.36.

3-(2-(Benzyloxy)-5-fluorophenyl)propan-1-ol (17)

Compound **17** was synthesized using the methodology as described for the preparation of similar compound in our previous work [3]. Thus, to a magnetically stirred solution of **16** (10.0 g, 41.27 mmol) in anhydrous THF (100 mL) was added a 0.5 M THF solution of 9-BBN (100.0 mL, 50.0 mmol) dropwise under a nitrogen atmosphere at 0 °C. The mixture was then stirred at room temperature for 6 h. The

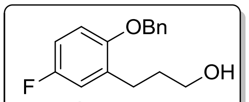

17

reaction was carefully terminated by the addition of H_2O (5 mL). Next, 3 N NaOH solution (50 mL) and 30% aqueous hydrogen peroxide solution (40 mL) were added to it sequentially. The reaction mixture was then stirred for an additional 2 h at 50 °C to complete the oxidation process. After cooling to rt, the mixture was extracted with ethyl acetate (2 × 100 mL), washed with water (150 mL) and brine (150 mL). The combined organic layers were dried over Na_2SO_4, filtered, and concentrated in a rotary evaporator. The crude product was purified by silica gel column chromatography (10% ethyl acetate in hexane) to afford **17** (10.31 g, 96%) as a colorless gum. ^{1}H NMR (400 MHz, $CDCl_3$): δ 7.45-7.33 (m, 5H), 6.92-6.84 (m, 3H), 5.05 (s, 2H), 3.60 (t, 2H, J = 6.4 Hz), 2.76 (t, 2H, J = 7.5 Hz), 1.89-1.82 (m, 3H). ^{13}C{^{1}H} NMR (100 MHz,$CDCl_3$): δ 157.1 (d, J_{C-F} = 238.7), 152.6, 136.7,132.3 (d, J_{C-F} = 7.7), 128.6, 128.0, 127.3, 116.8 (d, J_{C-F} = 23.0), 112.8 (d, J_{C-F} = 22.1), 112.7 (d, J_{C-F} = 7.7), 70.8 (t, J_{C-F} = 2.9), 61.6, 32.6, 26.0. Anal. Calcd. for $C_{16}H_{17}FO_2$: C, 73.83; H, 6.58. Found: C, 73.97; H, 6.69.

(*E*)-Ethyl 5-(2-(benzyloxy)-5-fluorophenyl)pent-2-enoate (18)

To an ice-cooled and stirred mixture of **17** (8.0 g, 30.73 mmol) and Celite® (4 g) in dry CH_2Cl_2 (100 mL) was added PCC (9.93 g, 46.10 mmol) under an atmosphere of nitrogen. The reaction was stirred vigorously for 3 h at rt. (Carbethoxymethylene)triphenylphosphorane (12.2 g, 35.0 mmol) was then added

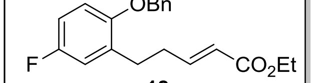

18

and the reaction was further stirred for 4 h at rt. After concentrating the reaction mixture in a rotary evaporator, the crude product was purified by silica gel column chromatography (4% ethyl acetate in hexane) to afford **18** (8.57 g, 85%) as a colorless oil. ^{1}H NMR (400 MHz, $CDCl_3$): δ 7.42-7.33 (m, 5H), 7.05-6.81 (m, 4H), 5.83 (d, J = 14.6), 5.06 (s, 2H), 4.19 (q, 2H, J = 7.3 Hz), 2.81 (t, 2H, J = 7.8 Hz), 2.52 (q, 2H, J = 7.8 Hz), 1.29 (t, 3H, J = 7.3 Hz). ^{13}C{^{1}H} NMR (100 MHz,$CDCl_3$): δ 166.6, 156.9 (d, J_{C-F} = 238.7), 152.5 (d, J_{C-F} = 1.9), 148.1,136.9, 131.3 (d, J_{C-F} = 7.7), 128.6, 127.8, 127.0, 121.7, 116.5 (d, J_{C-F}

= 23.0), 113.0 (d, J_{C-F} = 23.0), 112.5 (d, J_{C-F} = 8.6), 70.4 (t, J_{C-F} = 2.9), 60.1, 32.0, 28.9, 14.2. Anal. Calcd. for $C_{20}H_{21}FO_3$: C, 73.15; H, 6.45. Found: C, 73.23; H, 6.52.

(2*R*,3*S*)-Ethyl 5-(2-(benzyloxy)-5-fluorophenyl)-2,3-dihydroxypentanoate (19) and (2*S*,3*R*)-ethyl 5-(2-(benzyloxy)-5-fluorophenyl)-2,3-dihydroxypentanoate (20)

Diols **19** and **20** were synthesized using the methodology as described for the preparation of similar compounds in our previous work [3]. Thus, to a stirred solution of *tert*-butyl alcohol (30 mL) and water (35 mL) were added AD-mix-α (9.0 g) and methanesulfonamide (0.62 g, 6.44 mmol) at room temperature. The mixture was vigorously stirred at room temperature until both phases were clear and then cooled to 0 °C. A solution of cinnamate ester **18** (2.11 g, 6.44 mmol) in *tert*-butyl alcohol (5 mL) was added 0 °C. The reaction mixture was stirred at the same temperature for 24 h. The reaction was quenched at 0 °C by the addition of sodium bisulfite (10 g), warmed to room temperature, and further stirred for 1 h. The reaction mixture was then extracted with ethyl acetate (3 × 100 mL). The combined organic layers were washed with aqueous 2 N KOH solution (50 mL), water (50 mL), and brine (50 mL). After filtration, the filtrate was concentrated in a rotary evaporator. The crude product was purified by silica gel column chromatography (30% ethyl acetate in hexane) to afford **19** (2.07 g, 89%, ee not determined) as a colorless gum. $[\alpha]^{D}_{25}$ = -9.78 (c = 1.0, $CHCl_3$). ^{1}H NMR (400 MHz, $CDCl_3$): δ 7.43-7.31 (m, 5H), 6.94-6.83 (m, 3H), 5.05 (s, 2H), 4.29-4.23 (m, 2H), 4.07 (s, 1H), 3.90-3.87 (m, 1H), 3.22 (s br, 1H), 2.90-2.71 (m, 2H), 2.32 (s br, 1H), 1.96-1.87 (m, 2H), 1.29 (t, 3H, *J* = 7.3 Hz). ^{13}C{^{1}H} NMR (100 MHz,$CDCl_3$): δ 173.4, 157.0 (d, J_{C-F} = 238.7), 152.5 (d, J_{C-F} = 1.9), 136.9, 132.1 (d, J_{C-F} = 7.7), 128.5, 127.9, 127.1, 116.7 (d, J_{C-F} = 23.0), 112.8 (d, J_{C-F} = 22.1), 112.7 (d, J_{C-F} = 8.6), 73.2, 71.7, 70.7, 61.9, 33.6, 26.3, 14.0. Anal. Calcd. for $C_{20}H_{23}FO_5$: C, 66.29; H, 6.40. Found: C, 66.34; H, 6.48.

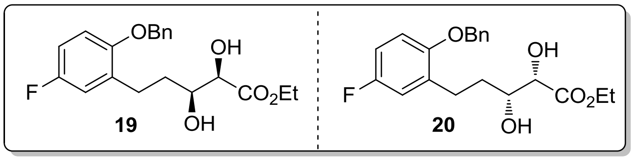

The corresponding (2*S*,3*R*) isomer **20** was synthesized using the same procedure as described above for the preparation of **19**, except using AD-mix-β (9.0 g) in place of AD-mix-α. Yield: 87% (2.02 g). $[\alpha]^{D}_{25}$ of **20** = +9.67 (c = 1.0, $CHCl_3$). ^{1}H NMR (400 MHz, $CDCl_3$) and ^{13}C{^{1}H} NMR (100 MHz,$CDCl_3$) data for **20** were identical with those of **19** (as described above).

Attempted cyclization via in situ-generated orthoester building block:

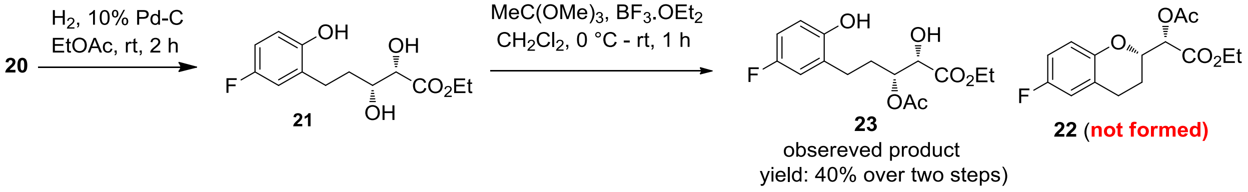

Debenzylation of **20** was achieved in a similar manner as described in our previous work [3]. Thus, to a stirred solution of **20** (200 mg, 0.55 mmol) in ethyl acetate (10 mL) was added 10% Pd–C (10 mg). After stirring for 2 h at room temperature under pressure of a hydrogen balloon, the reaction mixture was filtered through a pad of Celite® and the filtrate was concentrated under reduced pressure to get the corresponding phenolic derivative as a colorless semi-solid.

The above product was then dissolved in dry CH_2Cl_2 (10 mL). Trimethyl orthoacetate (54 μL, 0.58 mmol,) was added to it, followed by the addition of $BF_3{\cdot}Et_2O$ (6 μL, 0.048 mmol) at 0 °C. The reaction mixture was stirred at rt for 1 h, and quenched with aqueous acetone. The solvent was removed under reduced pressure, and the product was purified via column chromatography (10% ethyl acetate in hexane) to yield compound **23** as a colorless gum (49 mg, 40% over two steps). ^{1}H NMR (400 MHz, $CDCl_3$): δ 6.77-6.65 (m, 3H), 5.20-5.16 (m, 1H), 4.26-4.14 (m, 3H), 3.45 (s br, 1H), 2.63-2.58 (m, 2H), 2.10-1.96 (s, 5H), 1.25 (t, 3H, J = 6.87 Hz). ^{13}C{^{1}H} NMR (100 MHz,$CDCl_3$): δ 172.3, 170.2, 156.5 (d, $J_{C–F}$ = 237.7), 149.9 (d, $J_{C–F}$ = 1.9), 128.7 (d, $J_{C–F}$ = 6.7), 116.1 (d, $J_{C–F}$ = 23.0), 115.9 (d, $J_{C–F}$ = 7.7), 113.2 (d, $J_{C–F}$ = 22.0), 73.8, 71.5, 62.1, 30.1, 25.7, 20.6, 13.8.

(2*R*,3*S*)-Ethyl 5-(2-(benzyloxy)-5-fluorophenyl)-3-hydroxy-2-(tosyloxy)pentanoate (24) and (2*S*,3*R*)-ethyl 5-(2-(benzyloxy)-5-fluorophenyl)-3-hydroxy-2-(tosyloxy)pentanoate (25)

Compounds **24** and **25** were synthesized using the methodology as described for the preparation of similar compounds in our previous work [3]. Thus, to a stirred solution of **19** (200 mg, 0.552 mmol) in dry CH_2Cl_2 (50 mL) at 0 °C were added triethylamine (0.115 mL, 0.827 mmol) and TsCl (109 g, 0.571 mmol), successively. The reaction mixture was then kept in the refrigerator for 72 h. The reaction was quenched with a saturated aqueous NH_4Cl solution (20 mL) and extracted with CH_2Cl_2 (2 × 50 mL). The combined organic layers were washed with water (50 mL), brine (50 mL), and dried over anhydrous Na_2SO_4. After filtration, the filtrate was concentrated in a rotary evaporator to get the crude product which was rapidly passed through a small pad of silica gel to remove the front-line and base-line impurities to obtain **24** (242 mg, 85%) which was immediately used for the next step.

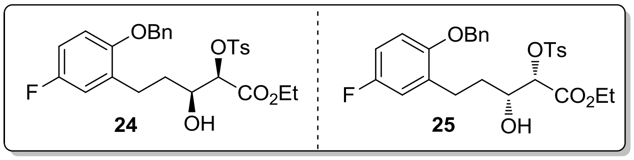

Compound **25** was synthesized using the same procedure as described above for the preparation of **24**, except using **20** (200 mg, 0.552 mmol) in place of **19**.

(*S*)-Ethyl 2-((*R*)-6-fluorochroman-2-yl)-2-hydroxyacetate (27) and (*R*)-ethyl 2-((*S*)-6-fluorochroman-2-yl)-2-hydroxyacetate (28)

To a stirred solution of **24** (242 mg, 0.47 mmol) in absolute ethanol (20 mL) was added 10% Pd–C (40 mg). The reaction was stirred at rt for 2 h under pressure of a hydrogen balloon. Then, K_2CO_3 (305 mg, 2.20 mmol) was added to the reaction mixture and the hydrogen balloon was replaced by a drying tube ($CaCl_2$). The reaction mixture was stirred for an additional 6 h at rt. The reaction mixture was filtered through a pad of Celite® and the filtrate was concentrated in a rotary evaporator and the filtrate was concentrated in a rotary evaporator. The resulting residue was dissolved in ethyl acetate (50 mL) and water (50 mL). The organic layer was separated, washed with brine (50 mL), dried over anhydrous Na_2SO_4 and filtered The organic layer was dried over Na_2SO_4, filtered, and concentrated in a rotary evaporator. Purification of the crude product by silica gel column chromatography (15% ethyl acetate in hexane) afforded **27** (83 mg, 70%) as a colorless semi-solid. $[\alpha]^D_{25}$ = -73.92 (c = 1.25, $CHCl_3$). ^{1}H NMR (400 MHz, $CDCl_3$): δ 6.79-6.68 (m, 3H), 4.37-4.28 (m, 3H), 4.25 (d, 1H, *J* = 1.8), 3.08 (s br, 1H), 2.93-2.77 (m, 2H), 2.18-1.96 (m, 2H), 1.32 (t, 3H, *J* = 6.9 Hz). ^{13}C{^{1}H} NMR (100 MHz,$CDCl_3$): δ 172.3, 155.6, 150.4, 122.7 (d, J_{C-F} = 7.7), 117.6 (d, J_{C-F} = 8.6), 115.1 (d, J_{C-F} = 22.0), 113.9 (d, J_{C-F} = 23.0), 76.4, 72.8, 61.9, 24.8, 23.2, 14.1. Anal. Calcd. for $C_{13}H_{15}FO_4$: C, 61.41; H, 5.95. Found: C, 61.56; H, 5.99.

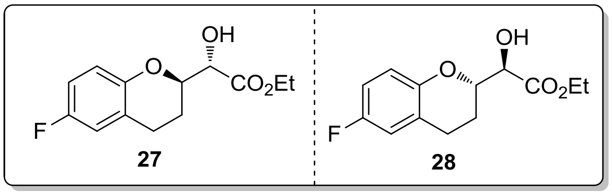

The corresponding (*R*,*S*) isomer **28** was synthesized using the same procedure as described above for the preparation of **27**, except using **25** (242 mg, 0.47 mmol) in place of **24**. Yield: 70% (83 mg). $[\alpha]^D_{25}$ of **28** = +73.78 (c = 1.0, $CHCl_3$). ^{1}H NMR (400 MHz, $CDCl_3$) and ^{13}C{^{1}H} NMR (100 MHz,$CDCl_3$) data for **28** were identical with those of **27** (as described above).

(*R*)-1-((*R*)-6-fluorochroman-2-yl)ethane-1,2-diol (2) and (*S*)-1-((*S*)-6-fluorochroman-2-yl)ethane-1,2-diol (29)

To a solution of **27** (200 mg, 0.786 mmol) in dry THF (10 mL) was added $LiAlH_4$ (77 mg, 1.96 mmol) portion wise at 0 °C under nitrogen atmosphere. After 1 h of stirring at room temperature, the reaction mixture was quenched by the addition of ethyl acetate (2 mL) and 1N HCl (5 mL) and then extracted with ethyl acetate (10 × 2 mL). The combined organic layers were washed with brine (20 mL), dried over Na_2SO_4, filtered and concentrated under reduced

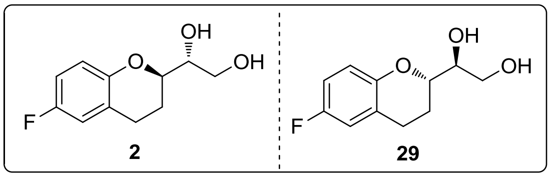

pressure. The crude product was purified by silica gel column chromatography (30% ethyl acetate in hexane) to afford **2** (155 mg, 93%) as a colorless solid. M.P.: 101-102 °C. $[\alpha]^{D}_{25}$ = +65.63 (c = 1.0, MeOH). Lit. $[\alpha]^{D}_{25}$ = +65.80 (c 1.0 MeOH) [4] and $[\alpha]^{D}_{20}$ = +63.08 (c 0.1, MeOH) [5]. ^{1}H NMR (400 MHz, $CDCl_3$): δ 6.83-6.73 (m, 3H), 4.09-4.04 (m, 1H), 3.85-3.77 (m, 3 H), 2.93-2.75 (m, 2H), 2.67 (s br, 1H), 2.13 (s br, 1H), 2.04-1.91 (m, 2H). Anal. Calcd. for $C_{11}H_{13}FO_3$: C, 62.26; H, 6.17. Found: C, 62.32; H, 6.25.

The corresponding (*S*,*S*) isomer **29** was synthesized in 93% yield using the same procedure as described above for the preparation of **2**, except using **28** (200 mg, 0.786 mmol) instead of **27**. $[\alpha]^{D}_{25}$ of **29** = -65.57 (c = 1.0, MeOH). ^{1}H NMR (400 MHz, $CDCl_3$) and $^{13}C\{^{1}H\}$ NMR (100 MHz,$CDCl_3$) data for **29** were identical with those of **2** (as described above).

(*R*)-1-((*S*)-6-fluorochroman-2-yl)ethane-1,2-diol (3)

To a stirred solution of DEAD (966 mg, 3.53 mmol) in THF (2 mL) were added sequentially solid PPh_3 (230 mg, 3.53 mmol), a solution of **29** (125 mg, 0.589 mmol) in dry THF (5 mL) and benzoic acid (431 mg, 3.53 mmol) in THF (5 mL) under an argon atmosphere at −10 °C. The resulting solution was stirred for 6 h at rt. It was then quenched with water (10 mL) and extracted with ethyl acetate (25 mL). The organic layer was washed with water and brine, dried over Na_2SO_4, filtered, and concentrated in a rotary evaporator. The residue was was subjected to silica gel column chromatography to remove most of the triphenyl phosphine oxide. The isolated product was not completely pure and still contained some impurities. Hence it was subjected to the next step without recording any spectral data.

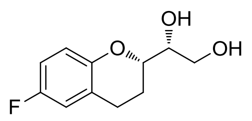

3

Similar as described in [3] to a solution of the resulting crude diester in MeOH (10 mL) was added a 10% MeOH solution of KOH (5 mL). The mixture was stirred for 2 h at rt. After removing acetone from the reaction mixture under reduced pressure, the resulting residue was dissolved in ethyl acetate (50 mL) and water (50 mL). The organic layer was dried over Na_2SO_4, filtered, and concentrated in a rotary evaporator. Purification of the crude product by silica gel column chromatography (30% ethyl acetate in hexane) afforded **3** (47 mg, 38% over two steps) as a colorless solid. M.P.: 86-87 °C. $[\alpha]^{D}_{25}$ = +71.39 (c = 0.2, MeOH). Lit. $[\alpha]^{D}_{25}$ = +71.80 (c 1.0 MeOH) [4] and $[\alpha]^{D}_{20}$ = +70.3 (c 0.1, MeOH) [5]. ^{1}H NMR (400 MHz, $CDCl_3$): δ 6.80–6.70 (m, 3H), 4.01–3.98 (m, 1H), 3.88-3.80 (m, 3H), 2.92 (s br, 1H), 2.87–2.74 (m, 2 H), 2.46 (s br, 1H), 2.15–2.11 (m, 1 H), 1.88-1.80 (m, 1 H). Anal. Calcd. for $C_{11}H_{13}FO_3$: C, 62.26; H, 6.17. Found: C, 62.38; H, 6.21.

(*S*)-6-Fluoro-2-((*R*)-oxiran-2-yl)chroman 5

Similar as described in [3] to a stirred solution of **28** (100 mg, 0.393 mmol) in dry CH_2Cl_2 (5 mL) at rt were added triethyl amine (0.075 mL, 0.654 mmol) and TsCl (74 mg, 0.393 mmol), successively. The reaction mixture was then stirred at rt for 12 h. The reaction was quenched with a saturated aqueous NH_4Cl solution (5 mL) and extracted with CH_2Cl_2 (3 × 15 mL). The combined organic layers were washed with water (10 mL), brine (10 mL), and dried over anhydrous Na_2SO_4. The solvent was removed under reduced pressure and the resulting residue was used for the next step without further purification.

To a solution of the obtained crude tosylate in THF (5 mL) was added 1 M $LiBH_4$ solution in THF (0.8 mL, 0.8 mmol) dropwise at 0 °C under nitrogen atmosphere. After 8 h of stirring at room temperature, the reaction mixture was quenched by the addition of ethyl acetate (2 mL) and 1N HCl (5 mL) and then extracted with ethyl acetate (10 × 2 mL). The combined organic layers were washed with brine (20 mL), dried over Na_2SO_4, filtered and concentrated under reduced pressure to give the corresponding hydroxy tosylate which was then dissolved in absolute ethanol (5 mL). K_2CO_3 (70 mg, 0.50 mmol) was added to it and the reaction mixture stirred vigorously for 10 h. After removal of ethanol under reduced pressure, the resulting residue *w*as redissolved in ethyl acetate (15 mL) and water (20 mL*).* The combined organic layers were washed with brine (20 mL), dried over Na_2SO_4, filtered and concentrated under reduced pressure. Purification of the crude product by silica gel column chromatography (15% ethyl acetate in hexane) afforded **5** (47 mg, 62% over two steps) as a colorless gum. $[\alpha]^{D}_{25}$ = +76.11 (c = 0.2, $CHCl_3$). Lit. $[\alpha]^{D}_{25}$ = +72.9 (c 1.0 $CHCl_3$) [6]. ^1H NMR (400 MHz, $CDCl_3$): δ 6.81-6.74 (3H, m), 3.85-3.81 (1H, m), 3.14-3.11 (1H, m), 2.90- 2.79 (4H, m), 2.16-2.11 (1H, m), 1.93-1.85 (1H, m). $^{13}C\{^1H\}$ NMR (100 MHz,$CDCl_3$): δ 156.8 (d, J_{C-F} = 237.9), 150.0, 122.8 (d, J_{C-F} = 7.3), 117.5 (d, J_{C-F} = 9.0), 115.2 (d, J_{C-F} = 21.8), 114.1 (d, J_{C-F} = 23.6), 75.5, 52.9, 45.7, 29.6, 24.2. Anal. Calcd. for $C_{11}H_{11}FO_2$: C, 68.03; H, 5.71. Found: C, 68.11; H, 5.78.

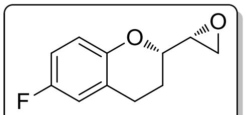

5

3. References

[1] Kitagawa, Y.; Sawada, H.; Kuchii, Y. Preparation of triazolopyrimidines as microbicides useful in agriculture and horticulture. PCT Int. Appl. WO 2002051845 A2 20020704, July 04, 2002.

[2] Choi, Y. M. Sulfamate derivatives and their preparation and use for the treatment of pain. PCT Int. Appl. WO 2015088272 A1 20150618, June 18, 2015.

[3] Das, S. K.; Panda, G. *Tetrahedron* **2008**, *64*, 4162-4173

[4] S. Chandrasekhar, M. V. Reddy, *Tetrahedron* **2000**, *56*, 6339–6344.

[5] N.-X. Wang, A.-G. Yu, G.-X. Wang, X.-H. Zhang, Q.-S. Li, Z. Li, *Synthesis* **2007**, 1154–1158

[6] Y.-X. Yang, S.-X. Liu, *J. Chem. Res*. **2007**, 506-508.

4. Copies of ^{1}H and ^{13}C NMR spectra of compounds

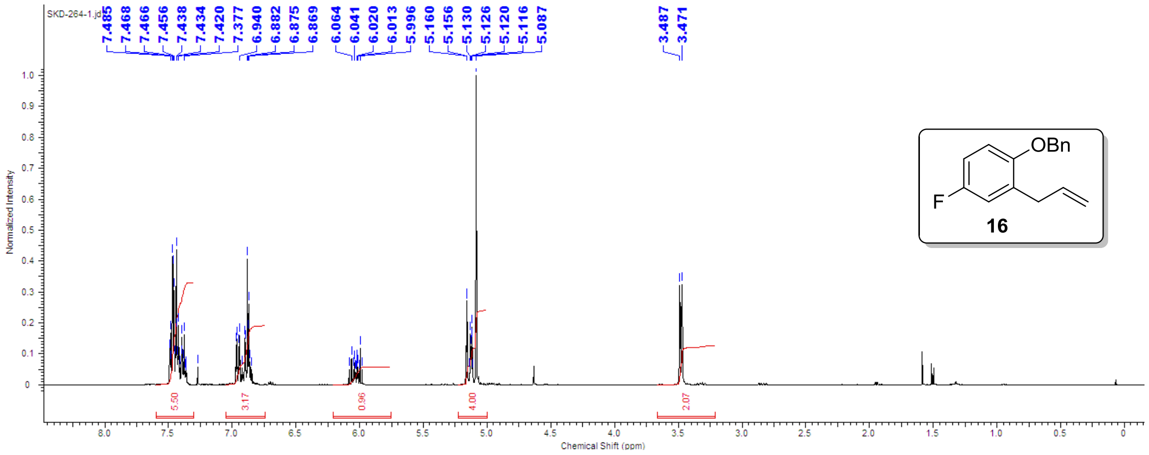

^{1}H NMR (400 MHz, $CDCl_3$) spectrum of compound **16**.

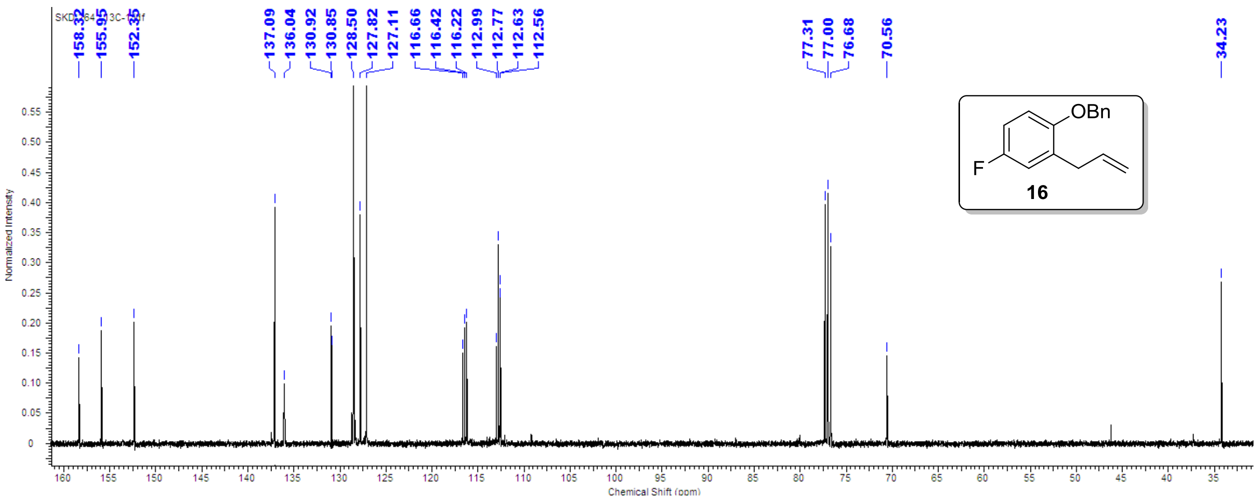

^{13}C NMR (100 MHz, $CDCl_3$) spectrum of compound **16**.

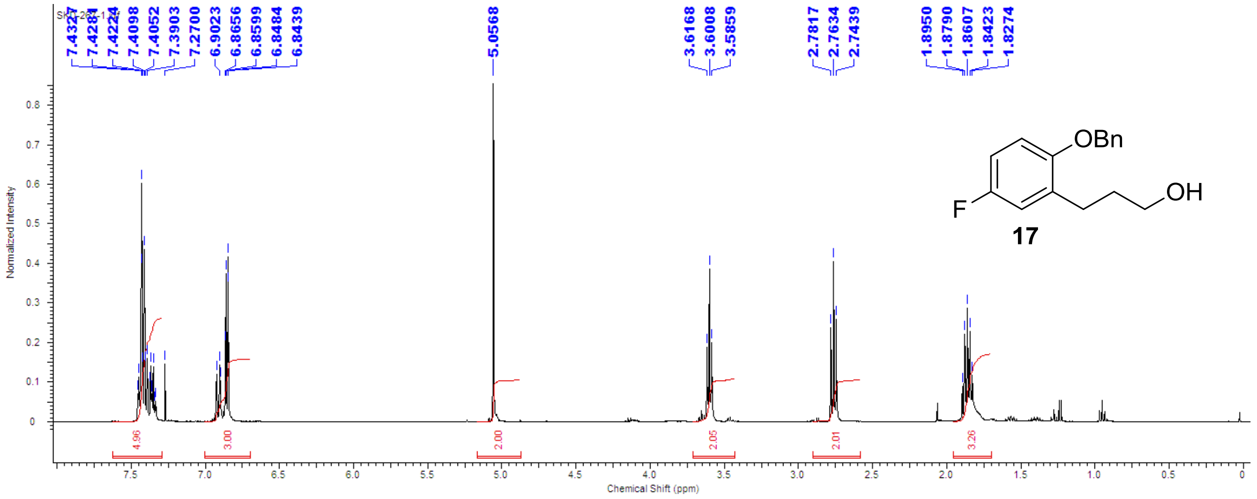

^{1}H NMR (400 MHz, $CDCl_3$) spectrum of compound **17**.

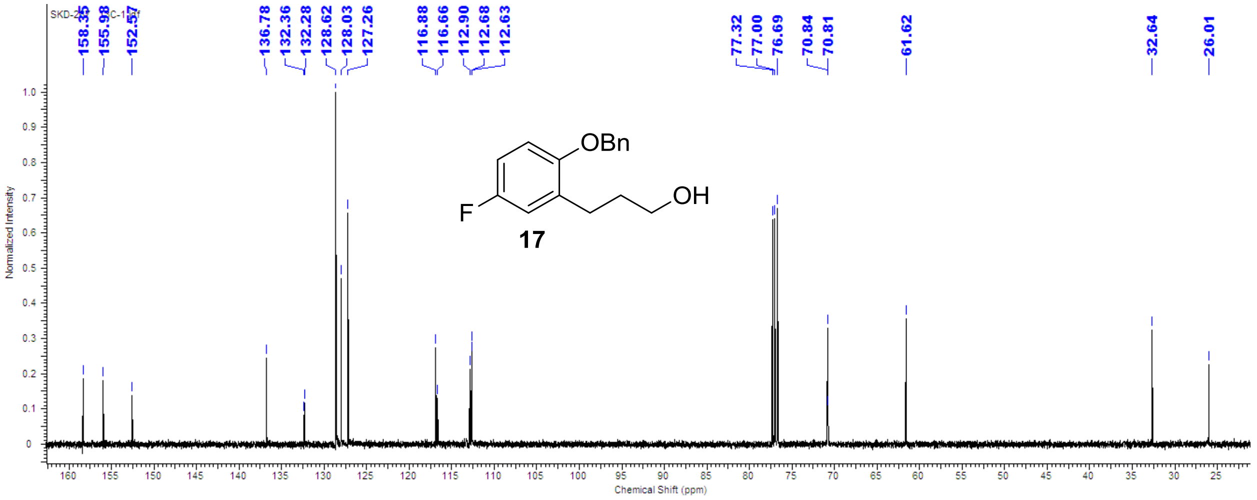

^{13}C NMR (100 MHz, $CDCl_3$) spectrum of compound **17**.

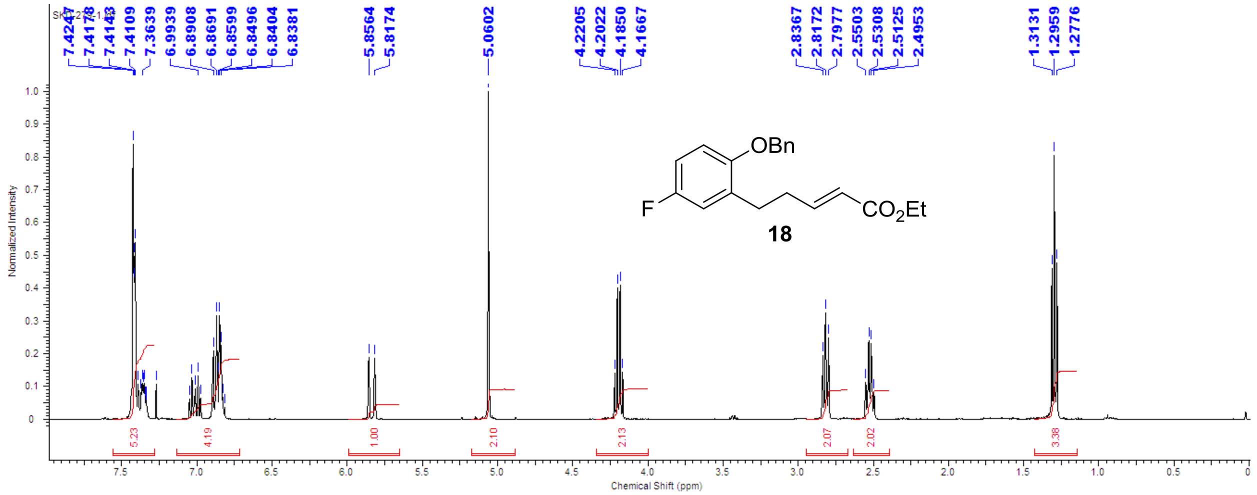

^{1}H NMR (400 MHz, $CDCl_3$) spectrum of compound **18**.

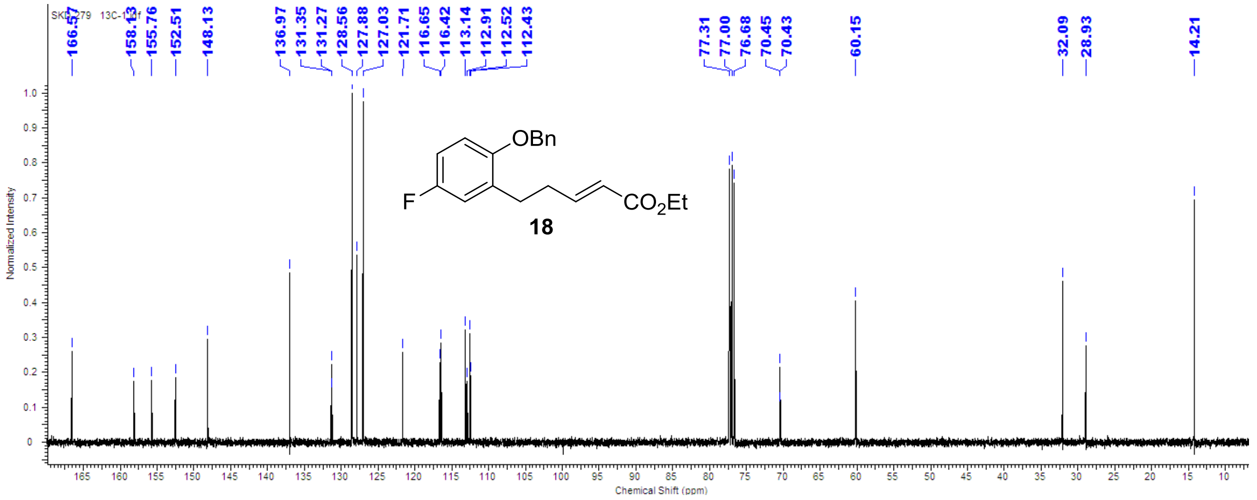

^{13}C NMR (100 MHz, $CDCl_3$) spectrum of compound **18**.

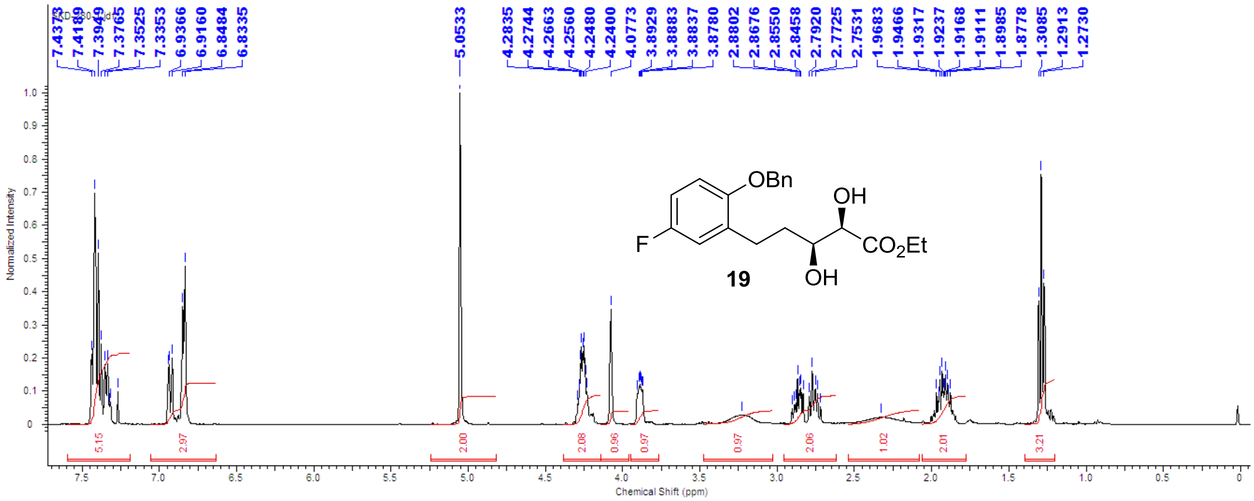

^{1}H NMR (400 MHz, $CDCl_3$) spectrum of compound **19**.

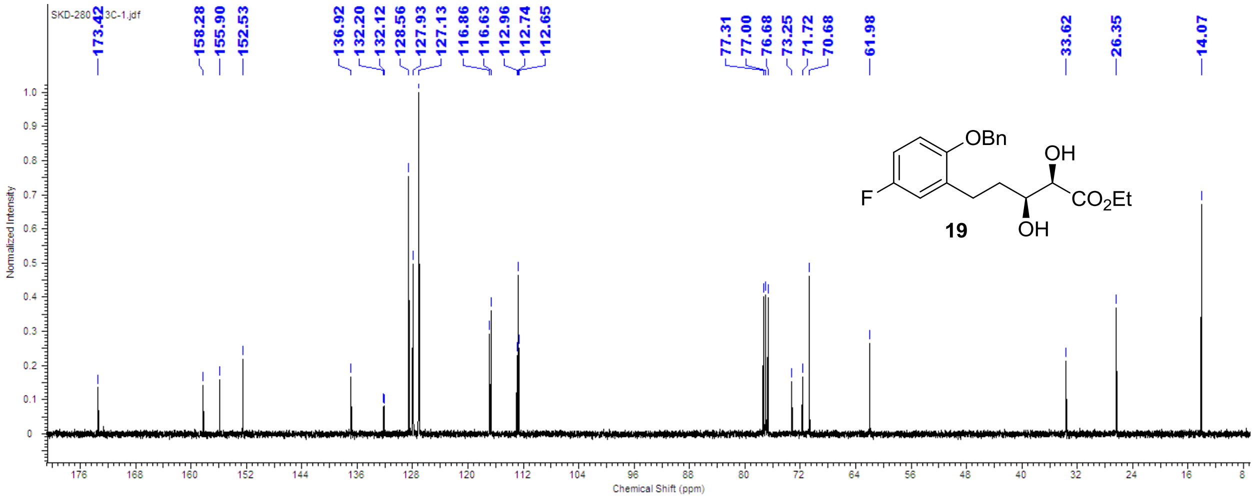

^{13}C NMR (100 MHz, $CDCl_3$) spectrum of compound **19**.

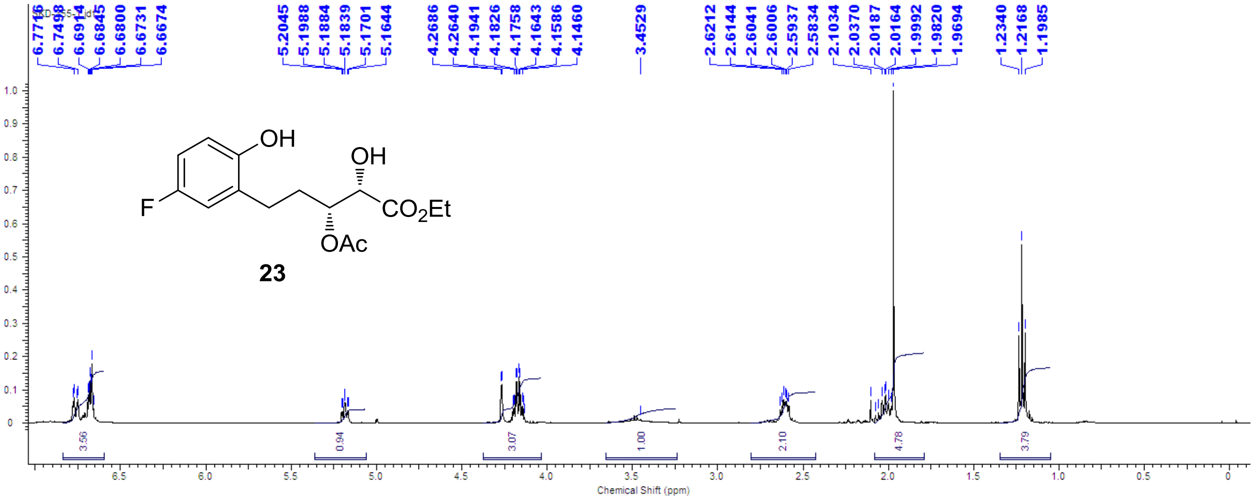

^{1}H NMR (400 MHz, $CDCl_3$) spectrum of compound **23**.

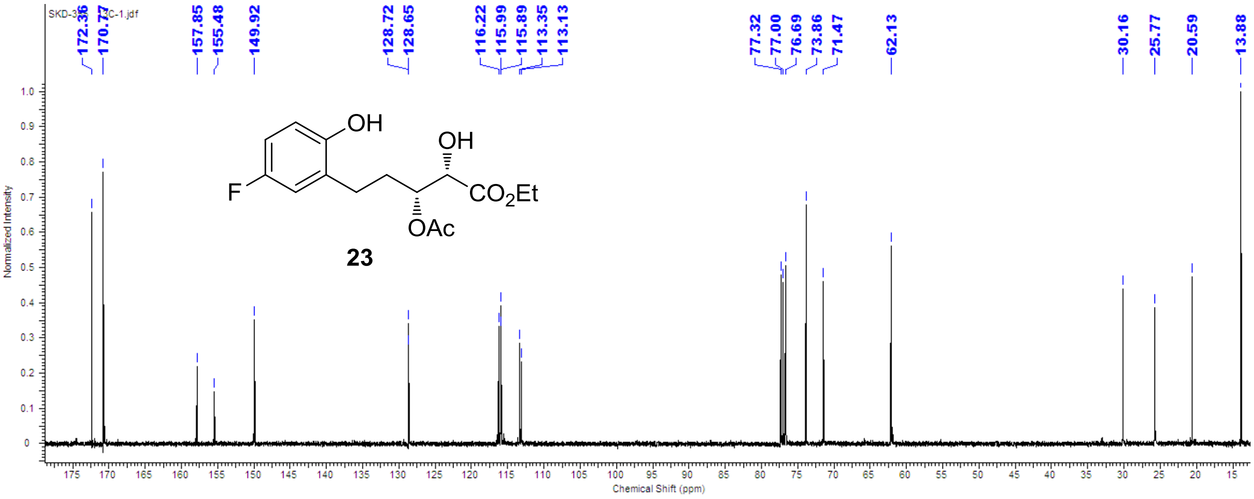

^{13}C NMR (100 MHz, $CDCl_3$) spectrum of compound **23**.

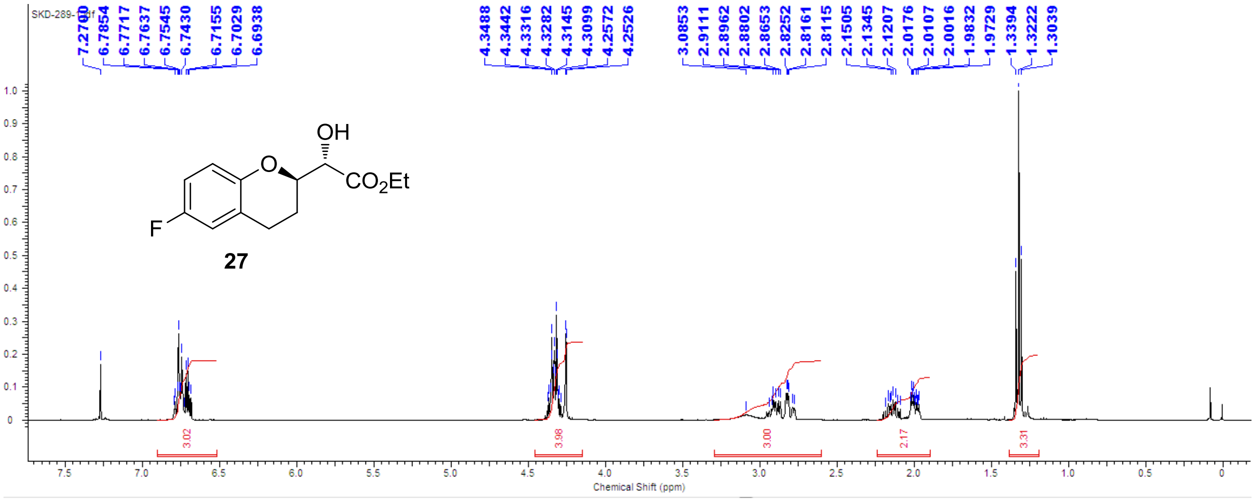

^{1}H NMR (400 MHz, $CDCl_3$) spectrum of compound **27**.

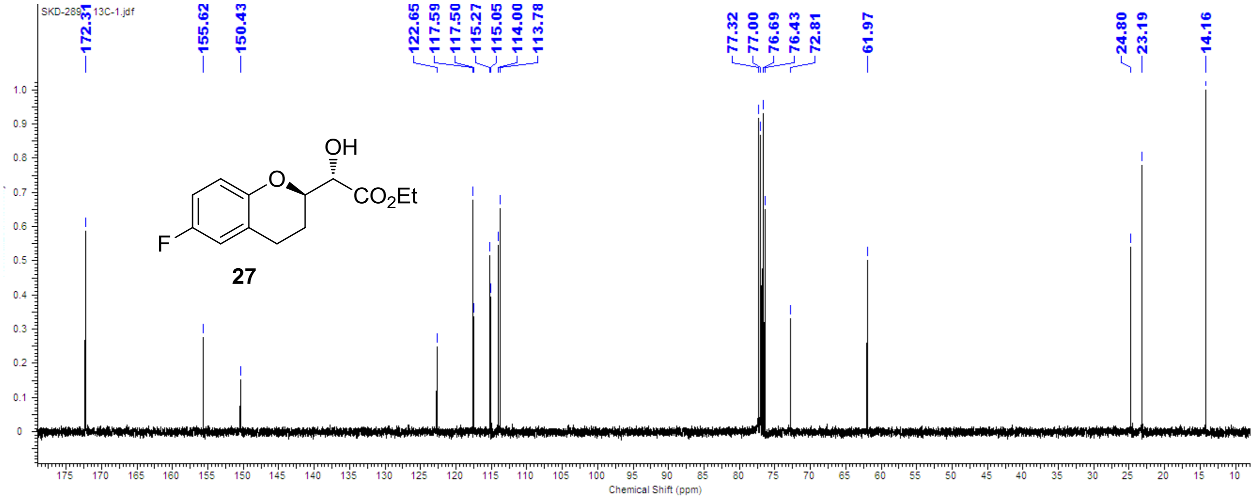

^{13}C NMR (100 MHz, $CDCl_3$) spectrum of compound **27**.

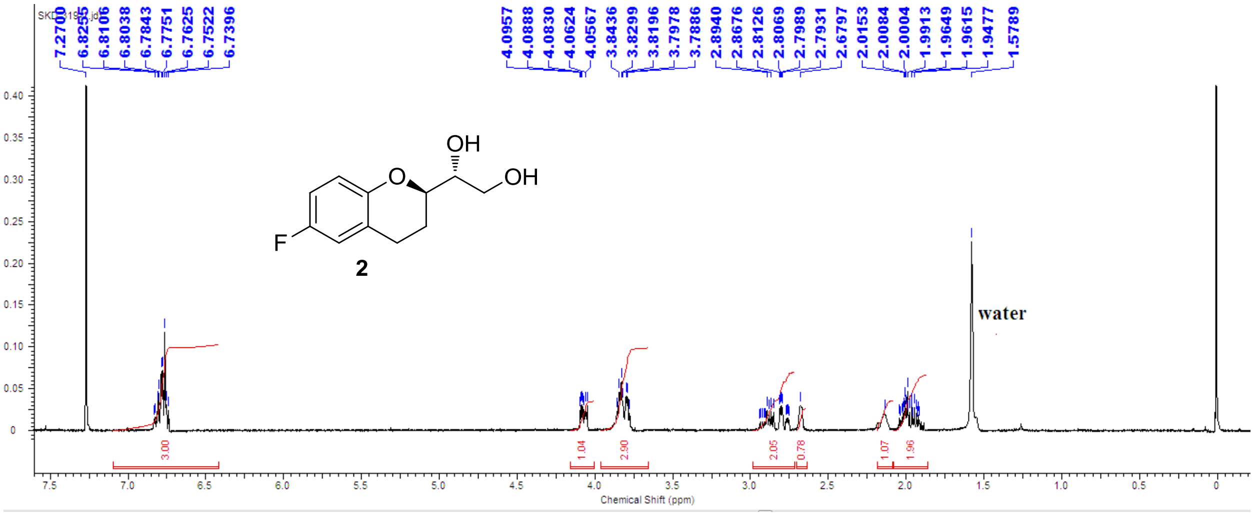

^{1}H NMR (400 MHz, $CDCl_3$) spectrum of compound **2**.

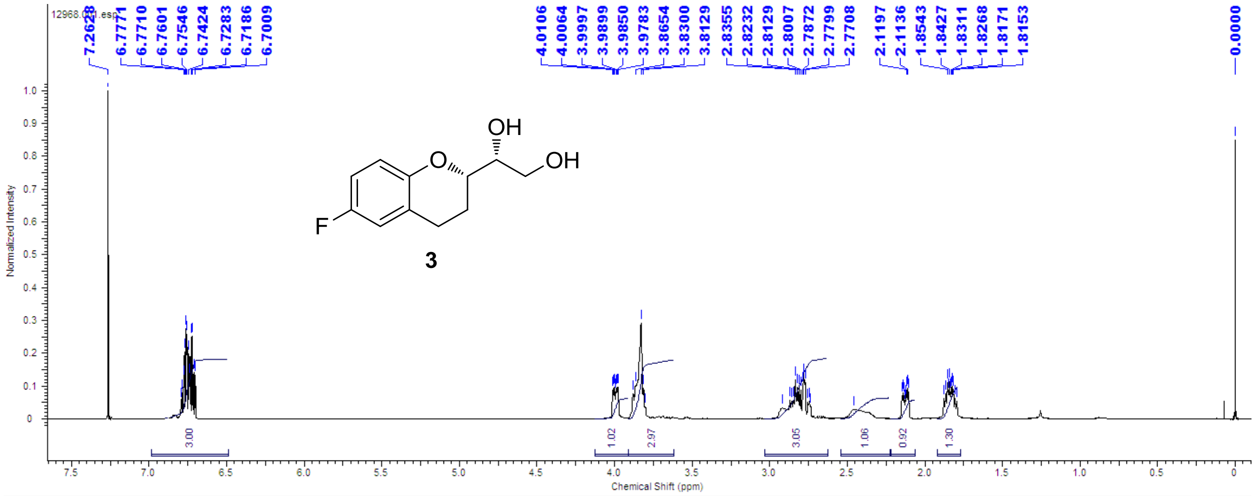

^{1}H NMR (400 MHz, $CDCl_3$) spectrum of compound **3**.

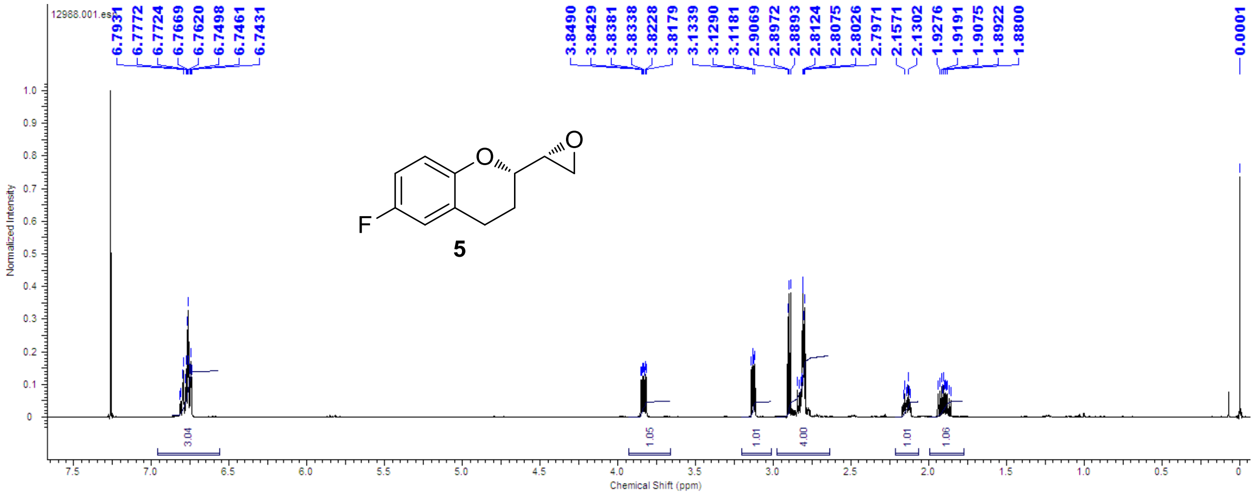

^{1}H NMR (400 MHz, $CDCl_3$) spectrum of compound **5**.

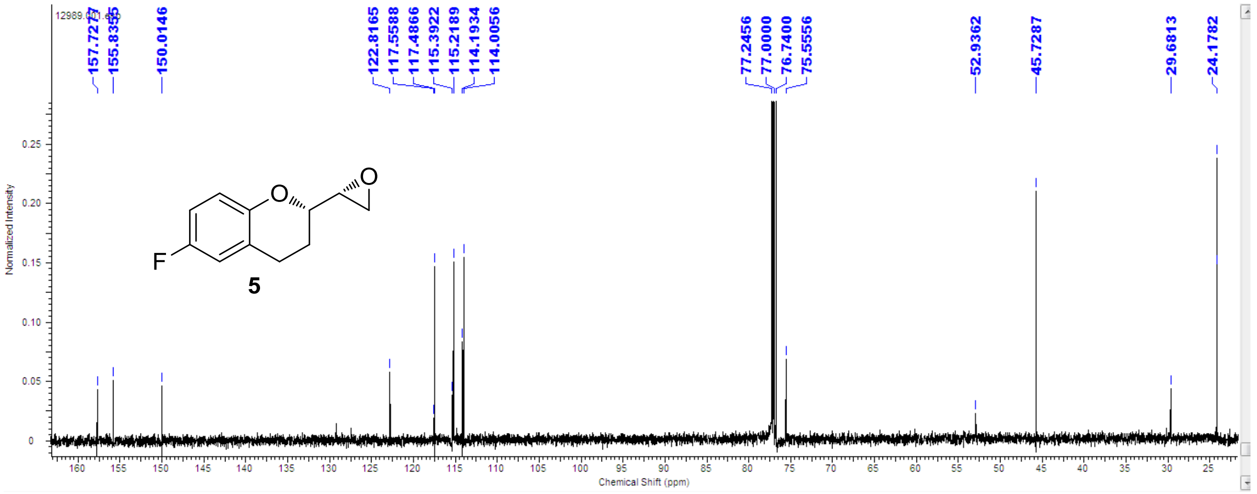

^{13}C NMR (100 MHz, $CDCl_3$) spectrum of compound **5**.
